# Assessing Knowledge, Practices, and Barriers to PrEP and nPEP Prescription Among Texas Healthcare Providers

**DOI:** 10.3390/healthcare12222315

**Published:** 2024-11-20

**Authors:** Yordanos M. Tiruneh, Ruchi Rachmale, Nagla Elerian, David L. Lakey

**Affiliations:** 1 Department of Preventive, Occupational, and Environmental Medicine, School of Medicine, The University of Texas at Tyler, Health Science Center, Tyler, TX 75708, USA; 2 Division of Infectious Diseases, Department of Internal Medicine, The University of Texas Southwestern Medical Center, Dallas, TX 75390, USA; 3 The University of Texas System, Austin, TX 78701, USA

**Keywords:** HIV, prevention, pre-exposure prophylaxis, post-exposure prophylaxis, PrEP knowledge, prescribing practice, healthcare providers, Texas, biomedical HIV prevention

## Abstract

Background: The effectiveness of pre-exposure prophylaxis (PrEP) and non-occupational post-exposure prophylaxis (nPEP) in preventing HIV is well-established, yet their use in clinical practice remains low. Healthcare providers, especially those in primary and emergency care settings, play a crucial role in adopting and implementing these prevention strategies. We conducted a statewide survey with 519 healthcare providers in Texas to assess their knowledge, practices, and barriers related to prescribing PrEP and nPEP. Methods: The survey collected data on demographics, clinical experience, practice type, awareness of recommended guidelines, knowledge of PrEP and nPEP, prescribing practices, and challenges encountered to prescribe these medications. We used multiple regression analysis to identify factors associated with PrEP and nPEP prescribing behavior. Results: While most providers were familiar with CDC and/or USPSTF-recommended PrEP guidelines, fewer had adequate knowledge of nPEP. Key challenges identified by providers included concerns about cost (48%), limited time (40% for PrEP and 51% for nPEP), and insufficient education or training (40% for PrEP and 35% for nPEP). Providers who were more familiar with the recommended guidelines and had greater experience in sexual health assessment were more likely to prescribe both PrEP and nPEP. Conclusions: This study highlights the need for enhanced education and training to boost providers’ knowledge and confidence in prescribing PrEP and nPEP. It also underscores the importance of addressing cost-related barriers and simplifying care processes to better integrate these HIV prevention strategies into primary and emergency care settings.

## 1. Introduction

With nearly 400,000 new cases each year, the human immunodeficiency virus (HIV) epidemic persists in the United States [1]. HIV, the causative agent of acquired immune deficiency syndrome (AIDS), poses a major global health challenge due to its widespread impact and complex prevention needs [2]. The Centers for Disease Control and Prevention (CDC) launched the Ending the HIV Epidemic (EHE): A Plan for America initiative in 2019, aiming to reduce new HIV infections by implementing scaled-up interventions and strategies, with a particular emphasis on biomedical interventions such as pre-exposure prophylaxis (PrEP) and non-occupational post exposure prophylaxis (nPEP) [3,4,5]. PrEP reduces the risk of acquiring HIV through daily or on-demand dosing schedules, while nPEP entails a 28-day course of antiretrovirals initiated within 72 h of possible exposure [1,6]. The CDC recommended nPEP in 2005, and it has been endorsed by the World Health Organization (WHO) to combat the global HIV epidemic [1].

The United States Preventive Services Taskforce (USPSTF) issued a Grade A recommendation for offering PrEP with effective antiretroviral therapy to those at high risk of acquiring HIV in 2013. However, PrEP use remains suboptimal in the United States [7,8]. As of 2020, approximately 25% of the 1.2 million individuals eligible for PrEP were prescribed it, making only a modest increase over five years [9,10]. PrEP is most frequently recommended for Black and Hispanic populations, yet these groups exhibit the lowest rates of PrEP utilization among the racial and ethnic demographics. According to data from the CDC, in 2020, only 9% (42,372) of the approximately 469,000 eligible Black individuals and only 16% (48,838) of nearly 313,000 eligible Hispanic individuals received PrEP prescriptions [8]. Similarly, the prevalence of PrEP use was lower in the southern states than in any other region, despite the South accounting for over half of newly diagnosed HIV cases in 2017. Only one-quarter of those prescribed PrEP were residing in the South [11,12].

An estimated 1.2 million individuals in the United States face a high risk of HIV, and achieving 40% PrEP uptake, based on CDC guidelines, could prevent around 33% of new infections in men who have sex with men (MSM) over a 10-year period [13]. A simulation study examining HIV epidemics in New York City revealed that up to a 23% reduction in new HIV infections could be achieved over a five-year period if 25% of high-risk MSM were to embrace PrEP [14]. These findings underscore the need for additional efforts to increase the adoption of biomedical HIV prevention interventions.

Promoting biomedical HIV prevention strategies necessitates the active engagement of healthcare providers to expand the use of PrEP and nPEP among HIV-vulnerable populations [1]. Recent data, however, suggest that physicians face obstacles when offering HIV prevention strategies. Many healthcare workers remain unfamiliar with PrEP and nPEP guidelines, lacking the knowledge and training needed to prescribe these interventions [1]. Although awareness and knowledge of PrEP has slowly increased since the publication of the CDC guideline, little is known about healthcare providers’ nPEP prescribing practices, despite its recommendation since 2005. Furthermore, a national study of nPEP awareness and prescribing practices in the US revealed that most providers who were “nPEP unaware” worked in the southern states [1]. This highlights regional disparities and emphasizes the need for targeted interventions in specific geographic areas to improve healthcare providers’ awareness and practices regarding both PrEP and nPEP.

To attain the EHE goal of reducing new HIV infections by 90% by 2030, it is imperative to enhance efforts in preventing new infections by systematically identifying major challenges [3]. In this survey, we sought to assess providers’ awareness of normative guidelines aimed at promoting biomedical HIV prevention practices and to identify challenges encountered when implementing these practices in Texas. Specifically, this survey delves into two key aspects: (1) awareness and prescribing practices related to oral PrEP (hereafter referred to as PrEP) and (2) awareness and prescribing practices concerning nPEP among medical providers with prescribing capabilities in Texas.

## 2. Materials and Methods

### 2.1. Study Design and Recruitment

We conducted a cross-sectional online survey using a convenience, non-probability sampling method targeting medical providers in Texas. The survey was distributed between mid-May and mid-July of 2021 through several professional associations, including the Texas Medical Association (TMA), the Texas Association for Community Health Centers (TACHC), and the Texas Hospitals Association (THA). Additionally, we distributed the survey via email and on the Texas Collaborative for HIV Education and Prevention (TCHEP) website. TCHEP is a collaborative funded by the Texas Department of State Health Services to promote the integration of HIV prevention efforts in primary and emergency care settings, guided by an advisory committee of local and state HIV prevention experts and advocates.

To encourage participation, periodic reminder messages were sent throughout the data collection period, and a 5 USD Starbucks gift card was offered as an incentive for survey completion. Upon opening the survey link, participants were directed to a participant information sheet and provided their consent by answering “yes” to a consent question. The study received ethical approval from the Institutional Review Board at the University of Texas Health Science Center at Tyler. Survey responses were anonymous, and participants who opted to receive the 5 USD gift card were directed to a separate link to provide their email address for the digital gift card.

### 2.2. Eligibility Criteria

The survey targeted medical providers licensed to prescribe in Texas, which includes medical doctors (MDs), doctors of osteopathy (DOs), nurse practitioners (NPs), and physician assistants (PAs). To participate in the survey, we asked a simple screening question: Are you a medical provider (MD, DO, NP, or PA) working in a primary or emergency care setting in Texas? The exclusion criteria included providers who were not practicing at the time of the study.

### 2.3. Questionnaire

We designed a 25-item survey to collect comprehensive data from the participants. The questionnaire was developed using elements from similar surveys, and we identified barriers from the literature review, then revised the contents based on input and feedback from the TCHEP Advisory Committee, which included a range of medical providers. The survey was administered electronically via the Qualtrics platform.

### 2.4. Measures

The primary outcomes of interest were the current prescribing practices of PrEP and nPEP, assessed through this question: “Do you currently prescribe PrEP/nPEP for patients who would benefit from these options?”. We collected demographic data, including age; sex; race (White, Black, Asian, more than one race, and Other); and ethnicity. Additionally, we gathered information on training and practice characteristics, such as years of clinical experience; medical specialty (family medicine, internal medicine, emergency medicine, obstetrics and gynecology, pediatrics, and others); site of medical practice; and type of practice. Awareness of recommended HIV prevention guidelines and intentions to prescribe PrEP/nPEP were also collected as independent variables. In addition, the survey examined beliefs about and perceived barriers to prescribing PrEP and nPEP in practice settings, with response options including (1) major barrier, (2) moderate barrier, (3) minor barrier, and (4) not a barrier at all.

### 2.5. Data Analysis

The data were downloaded from Qualtrics after the survey closed. Statistical analysis was performed using Stata SE 15.0 (StataCorp, College Station, TX, USA). Descriptive statistics summarized providers’ demographics, knowledge, and prescribing practices, as well as their beliefs, intentions, and barriers related to PrEP/nPEP for eligible patients. A complete case analysis was conducted. A multivariate logistic regression model was fitted to identify determinants for PrEP and nPEP prescriptions, controlling for sociodemographic factors, specialty, practice location, clinical characteristics, and knowledge of recommended guidelines. A *p*-value of less than 0.05 was deemed statistically significant.

## 3. Results

Of the 564 respondents who participated in the survey, 45 were excluded from the analysis due to failure to meet the eligibility criteria—either not working as an MD/DO, a PA, or an NP, or not practicing medicine at the time of the survey or failing to respond to the questions regarding PrEP and nPEP. The analysis was conducted with 519 respondents using a complete case analysis strategy, ensuring that each respondent’s completed responses were included in the analysis.

### 3.1. Demographic and Clinical Practice Characteristics

The respondents’ demographic characteristics are shown in Table 1. The median age was 47 years, with 264 (50.9%) identifying as female. Regarding ethnicity, 316 (61.6%) identified as White and 81% identified as non-Hispanic. Participants reported an average of 17 years of experience and were collectively providing care across 62 counties in Texas, covering 97 cities. The counties with the highest representation among the survey participants were Harris (21%), Travis (11.2%), Dallas (9%), Bexar (8.5%), and Hidalgo (4%).

Table 1 also provides statistics for practice specialties and work locations. Among the respondents, 501 (96.5%) were physicians, specializing in family medicine 190 (38.1%), internal medicine 111 (22.2%), pediatrics 78 (15.6%), emergency medicine, obstetrics and gynecology, and various other specialties, each comprising approximately 8% of participants. The majority of respondents were engaged in private medical practice 198 (39.5%) or worked at hospital- or university-affiliated clinics 157 (31.4%). Community Health Centers/Federally Qualified Health Centers (FQHC) and hospitals were reported as practice sites by 83 (16.6%) and 50 (9.9%) of the respondents, respectively. Approximately 383 (74.8%) of the clinicians were involved in primary care.

### 3.2. Knowledge of Recommended Guidelines

A majority of the providers who participated in the survey 356 (74.6%) were acquainted with the CDC and/or the USPSTF recommendations and guidance for prescribing PrEP to HIV-vulnerable patients, including partners of individuals living with HIV (Table 2). Among these respondents, 163 (34.2%) were actively prescribing PrEP for their patients, and more than half 260 (54.5%) had access to a referral system or network for PrEP providers or services. Nearly half 233 (49.3%) reported being likely to prescribe PrEP to individuals who could benefit from it. In contrast, a lower percentage of providers, 272 (58.2%), were familiar with CDC recommendations and guidance for prescribing nPEP following isolated, non-occupational high-risk events (such as sexual exposure or non-occupational instances of injection drug use) that might have exposed patients to HIV (Table 2). Only 142 (30.4%) of the providers reported prescribing nPEP.

### 3.3. Perceived Barriers to Prescribing Biomedical HIV Prevention Interventions

Among the respondents, 231 (48.5%) reported no barriers, 121 (25.4%) reported a minor barrier, and 124 (26%) reported a moderate or major barrier, reflecting unfamiliarity with CDC and USPSTF guidelines for prescribing PrEP (Table 3). Lack of training for prescribing and managing PrEP was reported as a major or moderate barrier by 192 (40.4%) of the providers, while 40.8% were concerned with the time required to provide counseling and monitoring for PrEP patients. Concerns about finances were rated as a major or moderate barrier by 227 (48%) of the providers, followed by knowing no medical providers who accept patients for PrEP services, 129 (27.2%). Perceptions that patients may not be interested in PrEP, 89 (18.8%), and that the patient population might not be vulnerable to HIV were reported by 67 (14.2%) as a major or moderate barrier to prescribing PrEP.

As is the case with PrEP, being unfamiliar with HIV prevention recommendations associated with nPEP, lack of training for assessing HIV-related sexual or injection-related exposure, or inadequate knowledge about prescribing nPEP were reported as moderate or major challenges to implementing nPEP recommendations by 181 (38.8%), 165 (35.4%), and 206 (44.5%) of the providers, respectively. Close to half, 224 (48.1%), of the providers reported issues related to lacking funds needed to cover costs as a major or moderate barrier to prescribing nPEP, while 203 (43.5%) reported concerns about follow-up care as a major or moderate barrier. Furthermore, 237 (51%) of the providers identified not seeing patients within the first 72 h following a high-risk event as a major or moderate barrier to prescribing nPEP, while less than one-fifth, 86 (18.5%), reported the perception that patients are not interested in nPEP as a major/moderate barrier.

Table 4 shows the results of a multivariable analysis examining prescribing practices for PrEP and nPEP in relation to specific provider characteristics. Pediatricians (aOR 0.1, *p* < 0.001) and providers specializing in obstetrics and gynecology (aOR 0.3, *p* = 0.028) were less likely to prescribe PrEP for their patients than providers specializing in internal medicine. Conversely, conducting sexual health assessments was significantly associated with higher odds that PrEP is prescribed (aOR 3.6, *p* = 0.003). Providers familiar with CDC and/or USPSTF recommendations and guidelines were 18 times more likely to prescribe PrEP (aOR 18.0, *p* < 0.001). For prescribing nPEP, providers who were familiar with CDC recommendations and guidelines were 37 times more likely to prescribe nPEP (aOR 37, *p* < 0.001) compared to those not familiar with those recommendations. No other provider or practice characteristics were found to be associated with prescribing nPEP.

## 4. Discussion

We have presented the findings of a survey conducted among medical providers in Texas who primarily engage with patients in primary and emergency care settings. This survey aimed to provide insights into their knowledge and practices concerning the delivery of biomedical HIV preventive care, PrEP, and nPEP. The study revealed three key findings. First, while a majority of clinicians demonstrated awareness of recommendations issued by normative bodies regarding PrEP administration, a smaller proportion was familiar with nPEP recommendations. Second, irrespective of their awareness of the recommendations, providers did not feel adequately trained to prescribe either PrEP or nPEP. Third, the study identified that both familiarity with PrEP and nPEP recommendations and experience in assessing the sexual health of patients were associated with a higher likelihood of prescribing both PrEP and nPEP.

While 75% of our respondents were familiar with the recommendations for prescribing PrEP to HIV-vulnerable patients, including the partners of HIV-positive individuals, fewer than 60% were acquainted with the guidance on prescribing nPEP following a non-occupational, isolated high-risk event that may have exposed the patient to HIV. Previous reviews highlighted reports in the literature indicating a lack of provider knowledge of PrEP [15,16]. Interestingly, primary care providers’ (PCPs’) awareness of PrEP increased from 24% to 66% between 2009 and 2015, but knowledge regarding PrEP remained low, with only 17% of providers reporting having read the CDC guidelines in 2014 [17]. In our study, over half of the respondents noted that a lack of familiarity with CDC and USPSTF guidelines was a barrier to prescribing PrEP and nPEP. This underscores the need for interventions that enhance providers’ knowledge of these prevention tools. These efforts are vital to meeting the 2030 goal of the EHE initiative. Solutions could include targeted training for healthcare providers on PrEP and nPEP, integrating HIV prevention resources into electronic health records, and creating support networks for providers, especially in underserved areas [3].

Many providers in our survey did not feel adequately trained to prescribe either PrEP (40%) or nPEP (35%). A lack of training specific to PrEP and nPEP among generalist providers may contribute to these results. Providers might perceive a lack of training due to the “purview paradox”, where PCPs believe that PrEP should be administered in HIV clinics due to a perceived lack of expertise in HIV medications, while HIV specialists believe primary care clinics are more suitable for PrEP provision as PCPs see more HIV-negative patients [15,16]. It stands to reason that this paradox could extend to nPEP administration as well. Expanding training programs, whether integrated into medical training or offered in continuing medical education (CME) courses, can empower providers with the confidence needed to prescribe PrEP and nPEP, particularly in rural areas facing resource scarcity.

Existing data suggest that targeted and contextually relevant training programs have proven highly effective in boosting providers’ knowledge and confidence in prescribing PrEP. For instance, after receiving presentations on PrEP, 13% of providers in a New York City study initiated PrEP prescriptions for the first time during follow-up visits [18,19]. Engaging primary care providers is crucial, especially considering that primary care fields including internal medicine, family medicine/general practice, and pediatrics comprise the highest numbers of active physicians in the United States and an estimated 1.2 million people could benefit from PrEP [19,20,21]. In New York City, a virtual training program in primary care and women’s health clinics, which integrated PrEP into routine STI management, led to a notable increase in PrEP prescriptions—from 11.5% to nearly half of providers [22]. Similarly, a PrEP optimization intervention that included a user-friendly online management tool and dedicated PrEP coordinator was also well received by healthcare providers, making it easier for them to incorporate PrEP into their practice [23].

On a positive note, while fewer than 40% of the providers in our study were currently prescribing PrEP for their patients, over half had the ability to access a referral system or network for PrEP providers or services for patients who request PrEP, indicating the available options for cases where direct care provision is challenging. Additionally, expanding telehealth services for PrEP prescription and management could further extend the reach of biomedical HIV prevention in rural and less populated counties. Incorporating PrEP training into the Project ECHO telementoring model proves to be a feasible and effective way to educate community healthcare providers, boosting their knowledge, confidence, and ability to stay updated on current PrEP guidelines [24]. Commercial services like NurX and Plushcare provide PrEP through virtual consultations with local or at-home testing coordination, though these services require further evaluation for impact on underserved groups. Experimental models such as PrEP@Home and ePrEP are assessing telehealth’s effect on PrEP uptake and maintenance, while hub-and-spoke systems—successfully used in southern regions for HIV treatment—could further support PrEP access by linking patients at Community Health Centers (CHCs) and Federally Qualified Health Centers (FQHCs) with remote specialists, thereby mitigating accessibility and stigma challenges [25,26,27].

Financial concerns about whether insurance or other payers will cover the cost of PrEP and nPEP emerged as a major concern in our survey, with 48% of respondents expressing apprehension, potentially impacting their willingness to prescribe these treatments. Similar findings were reported in a study of medical providers in Washington state, where 43% expressed concern about the cost of PrEP to patients [13]. Cost-related concerns encompass various factors, including the price of antiretrovirals, clinical visits, and laboratory testing, compounded by limited access to health insurance [28]. Among the 1.2 million US adults with PrEP indications in 2018, approximately 49,860 (4%) incurred uncovered PrEP-related costs. Of these individuals, only 3160 (6%) faced uncovered costs for both PrEP medication and associated clinical care, totaling 18.9 million USD, while the remaining 46,700 (94%) had uncovered costs solely for clinical visits and lab testing, amounting to 83.5 million USD. In total, the annual uncovered costs for adults with PrEP indications reached 102.4 million USD, highlighting the substantial financial burden despite the relatively low proportion (under 5%) of individuals with uncovered costs [28]. The average wholesale price of tenofovir disoproxil fumarate and emtricitabine (TDF-FTC)—the oral pill for PrEP—is approximately 1425 USD per month [17]. An analysis by Smith et al. estimated that nearly 90,000 people in the US faced costs for PrEP medication and care in 2015 that were not covered by insurance, so provider concerns are reasonable [29]. Pleuhs et al. posited, however, that providers’ lack of awareness of payment options for PrEP and nPEP, as well as insurance coverage options, may contribute to these concerns [15]. Under the Affordable Care Act (ACA), most private insurance plans and Medicaid expansion programs are mandated to cover PrEP [19]. Despite Texas not expanding Medicaid coverage, options such as state PrEP assistance programs and the federal Ready, Set, PrEP Program initiated in 2019 are available [30,31]. In addition, the emergence of multiple manufacturers and the introduction of a generic primary PrEP medication formula, Truvada, have contributed to reduced medication prices [18]. Increasing awareness of these developments could help providers in navigating financial challenges.

Concerns regarding the time commitment for treatment emerged as a notable barrier to prescribing PrEP in our study. More than 40% of providers expressed apprehension about the time required for counseling and monitoring of PrEP patients. Additionally, 51% reported difficulties in meeting the required 72 h or sooner timeframe for nPEP. A large-scale survey of physicians also identified the time needed for counseling patients and assessing adherence as a significant barrier [31]. Even providers specializing in HIV care acknowledged that time constraints hindered the delivery of effective care [32]. Streamlining follow-up processes by limiting certain visits to laboratory testing and involving ancillary staff in adherence counseling and risk reduction education can reduce demands on clinicians’ time. Patients requiring additional clinical attention can then be identified through targeted screening [33]. This approach ensures the delivery of high-quality care while promoting adherence to PrEP and nPEP protocols [34]. By optimizing operational efficiency, healthcare systems can better support providers in delivering timely and effective HIV prevention interventions.

In our study, we observed a significant association between awareness of CDC recommendations for PrEP and nPEP administration and the actual implementation of recommended HIV prevention practices. A national survey of primary care providers in 2015 found that only 66% reported being knowledgeable about PrEP. However, once PrEP was clearly defined, 91% of primary care providers expressed their willingness to prescribe it for high-risk patients and showed an interest in receiving relevant education [19,34]. These findings indicate a positive correlation between knowledge and providers’ willingness to engage in enhanced screening and prevention practices. Therefore, continuous efforts to improve education and training are crucial for fostering increased knowledge and promoting effective prevention strategies. To better equip healthcare professionals in HIV prevention, medical education should include training on nPEP and PrEP in both medical school and residency programs. Continuing education courses are also essential to help providers stay updated and confident in using the latest prevention recommendations [35]. By covering available PrEP options and clear guidelines for prescribing and follow-up, these programs can support providers in offering comprehensive, up-to-date HIV care.

Similarly, our study revealed that providers who actively assessed their patients’ sexual health were more likely to prescribe pre-exposure prophylaxis (PrEP). This finding aligns with other studies that have demonstrated a positive association between obtaining sexual health histories and implementing HIV screening practices [36,37]. Moreover, the prescription of nPEP has been linked to patient knowledge and requests [38]. Education focused on behavioral risk assessments, sexual history-taking, engaging in sensitive conversations, and patient-centered communication is essential to promote evidence-based HIV prevention practices. Offering continuing education courses will also help healthcare providers stay current with the evolving landscape of HIV transmission, diverse risk factors affecting different populations, and best practices in HIV prevention. Partnering with organizations that offer training in gender and sexual minority health can further enhance providers’ skills and understanding [19]. Further research could help identify specific training needs to make these programs as effective as possible.

Efforts to expand the adoption of PrEP and nPEP in Texas have been limited, underscoring the need to strengthen messaging and collaboration with state and local health departments, as well as professional associations. Effective strategies are essential to enhance providers’ understanding of nPEP and PrEP. Research indicates regional disparities in nPEP prescribing rates in the South, coupled with a low PrEP-to-need ratio (the number of PrEP prescriptions per reported HIV infection), which correlates with a higher HIV incidence [1]. Addressing these disparities requires comprehensive educational efforts and strategic policies to achieve a more robust implementation of PrEP and nPEP in Texas. Furthermore, qualitative studies are warranted to gain deeper insights into provider and structural challenges influencing the adoption of HIV prevention recommendations.

Policies should integrate precise guidelines into clinical practice and quality improvement programs. To enhance accessibility and affordability, providers need to be well informed about reimbursement mechanisms and insurance coverage, emphasizing the importance of incorporating such details into their practice [39]. Simultaneously, the expansion of community and public health entities could be prioritized to deliver targeted care. Collaboration with professional organizations is crucial for developing educational materials and online modules that can enhance provider knowledge [39]. Additionally, the incorporation of decision support tools and electronic health record (EHR) prompts can assist healthcare providers in adhering to recommended practices. Finally, it is crucial that medical education equips future clinicians with the knowledge and skills needed to incorporate biomedical HIV prevention strategies, like PrEP and nPEP, into their clinical practice. Integrating HIV prevention education into health professional school curricula through structured case-based modules and hands-on patient simulations has proven to improve providers’ readiness to assess risk, offer counseling, and manage preventive care effectively [35,40]. Targeted educational interventions, needs assessments, interactive teaching, and supplementary online resources, along with practical documentation tools, have proven successful in strengthening providers capabilities to adopt evidence-based practices. By adopting these measures, healthcare systems can enhance the uptake and effectiveness of PrEP and nPEP, ultimately improving HIV prevention outcomes and advancing the goal of ending the HIV epidemic [41].

This study is subject to certain limitations. First, our recruitment process involved reaching out to participants through various professional organizations in Texas. Since our survey relied on convenience sampling, there is a potential for selection bias, with providers more engaged in professional networks or affiliated with certain organizations potentially being overrepresented. As a result, our findings may not fully represent the diverse experiences and perspectives of all healthcare providers across Texas, which may limit the generalizability of the results. We believe, however, that the diversity of professional associations included in our study likely encompassed a broad spectrum of provider perspectives. Second, our study focused exclusively on providers in Texas, a state that did not expand Medicaid in conjunction with the ACA. Therefore, issues related to cost and insurance coverage of preventive services may not align with situations in states that expanded Medicaid. Nevertheless, our study sheds light on the experiences of providers in similar states that did not expand Medicaid, contributing valuable insights into the broader context of healthcare practices in these states.

## 5. Conclusions

This study conducted a comprehensive analysis of HIV prevention practices among medical providers in Texas, with a focus on their knowledge and implementation of PrEP and nPEP recommendations. The findings highlighted that many providers are aware of PrEP guidelines, but awareness of nPEP recommendations is lacking. Not surprisingly, there is a positive association between familiarity with recommended guidelines and practitioners’ likelihood to prescribe these preventive measures. The study revealed the multifaceted challenges influencing PrEP and nPEP prescriptions among healthcare providers in Texas, including barriers such as unfamiliarity with the guidelines, inadequate training to prescribe PrEP or nPEP, and concerns about costs and time. Our analysis underscores the importance of tailored interventions aimed at improving provider education and training in HIV prevention strategies, as well as addressing systemic barriers. Integrating HIV prevention education into health professional school curricula, using structured case-based modules and direct patient simulations, has been shown to enhance providers’ readiness to assess risk, counsel, and manage preventive care [35,40]. Recognizing and accommodating the diverse needs of different provider communities and demographics is also crucial for implementing effective public health interventions aimed at significantly advancing the goal of ending the HIV epidemic.

## Figures and Tables

**Table 1:** Sociodemographic and practice characteristics of medical providers.

Description/Characteristics	Category/Measure	Mean (SD) or Median (Q1–Q3) or N (%)
Age, years (n = 511)	Median (Q1–Q3)	47 (37–60)
	Mean (SD)	48.5 (14.2)
	Min–Max	23–82
Years of experience (n = 515)	Median (Q1–Q3)	15 (6–27)
	Mean (SD)	17.3 (13.3)
Gender	Male	243 (46.8)
	Female	264 (50.9)
	Prefer not to answer	12 (2.31)
Race (n = 513)	White	316 (61.6)
	Black	35 (6.8)
	Asian	109 (21.3)
	More than one race	22(4.3)
	Other	31 (6.1)
Hispanic/Latino	Yes	87 (16.8)
	No	419 (80.7)
	Prefer not to answer	13 (2.5)
Credentials	Medical Doctor (MD/DO)	501 (96.5)
	Physician Assistant	4 (0.77)
	Nurse Practitioner (NP, APRN, ARNP)	14 (2.7)
Medical Specialty (n = 499)	Family Medicine	190 (38.1)
	Internal Medicine	111 (22.2)
	Emergency Medicine	42 (8.4)
	Obstetrics & Gynecology	39 (7.8)
	Pediatrics	78 (15.6)
	Other Specialties	39 (7.8)
Site of Medical Practice (n = 507)	Private Medical Practice	198 (39.5)
	Community health center/ Federally Qualified Health Center (FQHC)	83 (16.6)
	Hospital-affiliated clinic (non-university affiliate)	61 (12.2)
	University-affiliated clinic	96 (19.2)
	Hospitals	50 (9.9)
	Other	19 (3.8)
Type of care offered (n = 517)	Primary Care	383 (74.8)
	Emergency Care	46 (8.9)
	Other	88 (17)

**Table 2:** Provider knowledge and practices related to HIV prevention strategies.

	Categories	N (%)
Are you familiar with the CDC and/or USPSTF recommendations and guidance for prescribing PrEP? (n = 477)	Yes	356 (74.6)
	No	91 (19)
	Not Sure	30 (6.3)
Are you currently prescribing PrEP for your patients? (n = 477)	Yes	163 (34.2)
	No	255 (53.5)
	Not Applicable	59 (12.4)
Do you or your clinic/practice have access to a referral system or network for PrEP providers? (n = 477)	Yes	260 (54.5)
	No	118 (24.7)
	Not Sure	99 (20.8)
How likely are you to prescribe PrEP to an individual who might be vulnerable to HIV or refer to PrEP services? (n = 473)	Very Unlikely	19 (4)
	Unlikely	49 (10.4)
	Likely	135 (28.5)
	Very likely	233 (49.3)
	Not Applicable	37 (7.8)
Are you familiar with CDC recommendations and guidance for prescribing nPEP? (n = 467)	Yes	272 (58.2)
	No	167 (35.8)
	Not Sure	28 (21)
Are you currently prescribing nPEP for patients who might have been exposed to a high-risk event of acquiring HIV? (n = 467)	Yes	142 (30.4)
	No	236 (50.5)
	Not Applicable	89 (19.1)

**Table 3:** Provider ratings of barriers to prescribing PrEP and nPEP in Texas.

Provider Ratings of Barriers to Prescribing PrEP	Major Barrier N (%)	Moderate Barrier N (%)	Minor Barrier N (%)	No Barrier at All N (%)
Providers rating of barriers to prescribing PrEP for patients who are vulnerable to HIV				
I am unfamiliar with the CDC and USPSTF guidance and recommendations for prescribing PrEP. (n = 476)	62 (13)	62 (13)	121 (25.4)	231 (48.5)
I am not trained to prescribe PrEP. (n = 475)	106 (22.3)	86 (18.1)	119 (25.1)	164 (34.5)
I am concerned about the time required to provide counseling and monitoring for PrEP patients. (n = 475)	80(16.8)	114 (24)	123(25.9)	158(33.3)
I am concerned about the finances to cover the cost of PrEP. (n = 475)	90 (19)	137 (28.8)	113 (23.8)	135 (28.4)
I don’t know any medical providers in our referral system who will accept patients for PrEP services. (n = 474)	64 (13.5)	65 (13.7)	93 (19.6)	252 (53.2)
My patients are not vulnerable to HIV. (n = 474)	17 (3.6)	50(10.6)	76(16)	331(69.8)
My patients are not interested in PrEP. (n = 473)	29 (6.1)	60 (12.7)	101 (21.4)	283 (59.8)
Provider ratings of barriers to prescribing nPEP				
I am unfamiliar with the prevention recommendations for nPEP. (n = 466)	105 (22.5)	76 (16.3)	111 (23.8)	174 (37.3)
I am not trained to assess for HIV-related sexual or injection-related exposures. (n = 466)	82 (17.6)	83 (17.8)	101 (21.7)	200 (42.9)
I am not trained to prescribe nPEP. (n = 464)	119 (25.7)	87 (18.8)	102 (22)	156 (33.6)
I am concerned about the finance to cover the cost of nPEP. (n = 466)	94 (20.2)	130 (27.9)	102 (21.9)	140 (30)
I am concerned about the follow-up required to provide counseling and monitoring for patients on nPEP. (n = 466)	78 (16.7)	125 (26.8)	117 (25.1)	146 (31.3)
I don’t see patients within the required 72-h or sooner time range. (n = 465)	124 (26.7)	113 (24.3)	103 (22.2)	125 (26.9)
My patients are not interested in nPEP. (n = 466)	28 (6%)	58 (12.5)	120 (25.8)	260 (55.8)

**Table 4:** Logistic regression model of PrEP and nPEP prescribing practices by provider and practice characteristics in Texas.

	Prescribing PrEP N = 346		Prescribing nPEP N = 335	
Provider Characteristics	OR (95%CI)	*p*-Value	OR (95%CI)	*p*-Value
Age	1.0 (0.9–1.0)	0.140	1.0 (0.99–1.0)	0.663
Years	1.1 (0.99–1.1)	0.070	1.0 (0.98–1.0)	0.262
Gender				
Male	(ref)		(ref)	
Female	1.0 (0.6–1.8)	0.960	0.8 (0.4–1.5)	0.480
Prefer not to Answer	0.6 (0.05–8.3)	0.732		
Race				
White	(ref)		(ref)	
Black	0.9 (0.3–2.5)	0.784	0.5 (0.2–1.7)	0.276
Asian	0.7 (0.3–1.5)	0.393	0.7 (0.3–1.6)	0.408
More than one race	1.1 (0.3–5.2)	0.860	3.9 (0.8–18.4)	0.088
Other	1.4 (0.4–5.5)	0.617	1.2 (0.2–7.0)	0.822
Hispanic/Latino				
No	(ref)		(ref)	
Yes	1.1 (0.6–2.5)	0.754	0.6 (0.3–1.6)	0.352
Prefer not to state	2.7 (0.1–134.3)	0.615		
Medical Specialty				
Internal Medicine	(ref)		(ref)	
Family/General/Preventive Medicine	0.9 (0.4–1.7)	0.709	0.97 (0.4–2.1)	0.957
Emergency Medicine	1.8 (0.2–20.1)	0.637	4.5 (0.7–30.4)	0.122
Pediatrics	0.1 (0.03–0.3)	0.000	0.8 (0.3–2.4)	0.709
Obstetrics & Gynecologyy	0.3 (0.1–0.9)	0.028	0.7 (0.2–2.4)	0.578
Other Specialties	0.7 (0.2–2.7)	0.644	0.7 (0.2–3.0)	0.670
Practice				
Private Medical Practice	(ref)		(ref)	
Community Health Center or Federally Qualified Health Center	0.8 (0.3–1.7)	0.493	0.8 (0.3–2.0)	0.656
Hospital-Affiliated Clinic (non-University)	1.1 (0.4–2.9)	0.871	0.8 (0.3–2.4)	0.681
University-Affiliated Clinic	0.7 (0.3–1.5)	0.363	0.6 (0.3–1.4)	0.267
Hospital	0.3 (0.1–1.2)	0.083	0.9 (0.2–3.7)	0.934
Other	0.4 (0.1–1.5)	0.171	1.0 (0.2–4.9)	0.996
Type of Care Offered				
Primary Care	(ref)		(ref)	
Emergency Care	0.3 (0.02–2.8)	0.261	1.6 (0.3–9.7)	0.589
Other	2.3 (0.9–6.1)	0.089	0.7 (0.3–1.9)	0.505
Sexual Health Assessment				
No	(ref)		(ref)	
Yes	3.6 (1.5–8.5)	0.003	2.4 (0.9–6.1)	0.072
County of Medical Practice				
Counties with the largest population *	(ref)		(ref)	
Counties with lesser population	0.7 (0.4–1.1)	0.140	0.7 (0.4–1.3)	0.298
Familiar with the CDC and/or USPSTF recommendations and guidance for prescribing PrEP				
No	(ref)			
Yes	18.0 (5.1–63.4)	0.000		
Familiar with the CDC recommendations and guidance for prescribing nPEP				
No			(ref)	
Yes			37 (12.8–106.9)	0.000

* Texas counties with the largest populations include Dallas, Harris, Travis, Tarrant, and Bexar. To avoid collinearity, provider credentials were excluded from the model.

## Data Availability

The original contributions presented in the study are included in the article, further inquiries can be directed to the corresponding author/s.
